# Experimental Evaluation of a Braille-Reading-Inspired Finger Motion Adaptive Algorithm

**DOI:** 10.1371/journal.pone.0148356

**Published:** 2016-02-05

**Authors:** Melda Ulusoy, Rifat Sipahi

**Affiliations:** Department of Mechanical and Industrial Engineering, Northeastern University, Boston, MA, United States of America; Eberhard Karls University of Tuebingen Medical School, GERMANY

## Abstract

Braille reading is a complex process involving intricate finger-motion patterns and finger-rubbing actions across Braille letters for the stimulation of appropriate nerves. Although Braille reading is performed by smoothly moving the finger from left-to-right, research shows that even fluent reading requires right-to-left movements of the finger, known as “reversal”. Reversals are crucial as they not only enhance stimulation of nerves for correctly reading the letters, but they also show one to re-read the letters that were missed in the first pass. Moreover, it is known that reversals can be performed as often as in every sentence and can start at any location in a sentence. Here, we report experimental results on the feasibility of an algorithm that can render a machine to automatically adapt to reversal gestures of one’s finger. Through Braille-reading-analogous tasks, the algorithm is tested with thirty sighted subjects that volunteered in the study. We find that the finger motion adaptive algorithm (FMAA) is useful in achieving cooperation between human finger and the machine. In the presence of FMAA, subjects’ performance metrics associated with the tasks have significantly improved as supported by statistical analysis. In light of these encouraging results, preliminary experiments are carried out with five blind subjects with the aim to put the algorithm to test. Results obtained from carefully designed experiments showed that subjects’ Braille reading accuracy in the presence of FMAA was more favorable then when FMAA was turned off. Utilization of FMAA in future generation Braille reading devices thus holds strong promise.

## Introduction

Braille literacy is crucial for blind individuals, as it enables life-long learning and is key to employment and independency [1]. One way to promote Braille literacy is to make existing Braille reading devices more accessible, affordable, and user friendly. Commercially available Braille reading devices in this regard need various improvements [2, 3]. Most of these devices cost thousands of dollars, mainly because they rely on multiple piezoelectric actuators in order to create the Braille letters [3]. Other issues include high voltage actuation and reduced portability. In order to solve these issues and improve existing Braille displays, various actuation methods such as thermal, electrical, and mechanical, have been widely investigated [4–9].

Among existing actuation techniques, electroactive polymer (EAP) materials have recently gained attention on the development of sheet type Braille displays [10, 11], where tiny actuators are packed in array form to produce refreshable Braille. Although these actuators hold promise for creating full page Braille displays, improvements are needed especially by lowering their voltage requirements, enhancing their bandwidth and increasing generated forces [12]. Other studies aim to reduce the high cost of conventional displays by designing Braille displays comprising a single Braille cell. In [13], a single cell has been mounted on a movable carriage, where Braille is displayed by a lateral wave of up and down moving pins, but no human subject experiments have been reported. Another study [14] also uses a single Braille cell, which is placed at the end effector of a planar Pantograph. The user reads a page by moving the single cell over the workspace of the haptic device. However, this task yields lower reading speeds when compared with conventional displays. In a more recent study [15], authors developed a novel Braille display by stimulating the skin laterally instead of using normal indentation. High legibility rates are reported from experiments with experienced Braille readers. However, numbness was noted in the reading fingers, and this virtual Braille display is reported to be more demanding and error prone when compared to conventional Braille displays.

Although a design approach based on single cell Braille display addresses the cost and size issues, having the finger rest on a Braille letter is undesirable. This is mainly because while reading with a single Braille cell, the sweeping motion of the finger over the Braille letters is lost. This brushing motion of the finger across Braille characters plays an important role in fine tactile sensing and Braille reading [16, 17]. Due to this reason and other issues such as low reading speeds and fatigue, it is not possible to create an effective Braille reading device with only a single cell display. While studies in this field still focus on alternative actuation methods to solve the cost and size issues, to the best of our knowledge there has been no study investigating the aforementioned problems with a focus of users’ needs, namely by taking into account the finger dynamics during Braille reading.

One of the main challenges in designing a versatile Braille reading device is because Braille reading is a complex process. Study of finger dynamics in this context has attracted considerable attention. In [18], the effects of letter position coding during Braille word recognition are investigated, where Braille reading is performed using a commercially available refreshable Braille display allowing detection of the finger position. The study in [19] investigates different characteristics of finger motion during Braille reading by making use of a digitizing tablet to record finger positions. Authors in [20] propose a finger-tracking system that utilizes a refreshable Braille reading display with a Wii Remote. Indeed finger dynamics and especially finger-rubbing actions across Braille letters is the key element in successful Braille reading as it promotes the stimulation of appropriate nerves for accurate reading. Moreover, it is known that such finger actions present intricate patterns across the letters both from left to right and right to left. The right-to-left movement of the finger, referred as “regression” [21] or “reversal,” [22] is crucial as it allows one to re-read the letters that were missed in the first pass, and can be performed by users as often as in every sentence, originating at any location in a sentence. While no reading takes place during a reversal action, reversals are one of the main characteristics of Braille reading, and are performed even by the most experienced Braille readers [19].

The above observations support the fact that a Braille reading device must be adaptable to users’ reading patterns, and it is not trivial to reduce costs associated with increased number of actuators by designing a single cell Braille display. Consequently, here, we envision that it would be useful if a Braille reading device could in real-time sense a user’s finger gestures and adjust Braille refresh rate accordingly to accommodate reversals as well as any speed changes the user voluntarily chooses during reading. While such a device currently does not exist, one prototype device that has the potential to achieve this is the Rotating Wheel Braille Display [23]. This display, which is not available in the commercial market, was built to create continuous reading experience by configuring the Braille letters around a rotating drum. Another advantage of it is that it requires only fewer actuators in a small compact volume, with drum diameter 5–10 cm, compared to other existing devices, holding promise toward an affordable and portable device. Nevertheless, this prototype, although designed to move bi-directionally, operates at constant speeds only [24] and hence does not accommodate reversals. Moreover, performance assessment of the device is not available as it was not thoroughly studied in human subjects experiments but only in preliminary piloting, which showed strong promise for further expansion. Therefore, we believe that an ideal Braille display device could be one that has been validated by extensive human subjects testing, and that tailors a drum-like Braille display, similar to the rotating wheel Braille display, with wheel speeds that can automatically adapt to user’s finger motion, in real-time.

In light of the above rationale, a finger motion adaptive algorithm (FMAA) has been developed by the authors. In order to access the feasibility of FMAA, we first study it with sighted subjects in experiments comprising a mechanical setup similar to the rotating wheel Braille display, and a touchscreen monitor. This algorithm does not need any sensors to be attached to the users, and functions effectively only by estimating variations in certain physical variables in the mechanical setup and the touchscreen monitor, specific to how the user’s finger interacts with the machine/device. To maintain the connection of this study with Braille reading, Braille-analogous tasks were designed requiring sighted subjects to perform certain “tracking” activities by interacting with the machine/device with their fingers at speeds consistent with Braille reading. In the design of these tasks, two criteria are taken into account: *a*) task performance must be measurable via some metrics such that differences, if any, with and without FMAA can be studied, *b*) tasks should require similar horizontal finger motion as in Braille reading including backward direction, to account for reversals.

Based on the performance criteria describing task completion percentage and tracking accuracy in the experimental setups, we find that the associated performance metrics were significantly different between two comparison groups in balanced experiments, namely, with and without FMAA, in favor of subjects utilizing FMAA. Results suggest that subjects took advantage of FMAA to improve their performance metrics, compared to tasks that were performed without the presence of FMAA. Moreover, subjective NASA TLX results indicate that the proposed algorithm increased mental workload of subjects. This, in connection with improvements in subjects’ performance, indicates that the algorithm successfully engaged the users with the tasks, while achieving a fine balance between mental workload and performance [25]. Additional discussions and insights gained from the experiments are also provided following the statistical analysis.

Promising results obtained with sighted subjects encouraged authors to next perform preliminary Braille reading tasks. In these experiments, FMAA is used to estimate in real-time the frictional forces applied by the user reading Braille wrapped around a rotating wheel, by monitoring how much the wheel tends to slow down, or accelerate. This information is then used to regulate wheel speed accordingly [26] to deliver the user a comfortable Braille reading speed. Carefully designed Braille reading tasks are tested by five blind volunteers to assess the effectiveness of FMAA in terms of Braille reading accuracy. Similar outcomes as in the experiments with sighted people are obtained in terms of efficacy of FMAA. Experimental results showed that subjects achieved higher reading accuracy rates while FMAA was on, compared to when FMAA was off.

While this study is based on Braille-reading-inspired tasks, it also provides contributions to the utilization of touchscreen monitors as part of man-machine systems [27, 28]. To the best of our knowledge, a touchscreen system with a dynamic adaptation nature to human touches in the closed-loop-control sense has not been so far reported in the literature although some studies already investigated eye-gaze control on various tablets [29–31]. Recent studies along these lines in the “open loop” sense include gaming in computer science [32–34], human cognition in neuroscience [35, 36], as well as rehabilitation [37, 38]. While in some of these studies, users need to adapt to the system, in some others, the term “adaptive” refers to a system whose parameters can be adjusted by the user during operation in an “open” loop sense but not automatically. A more desirable adaptive system such as the one proposed here would dynamically and automatically adapt to user’s needs in real-time, without having to require the user to make the necessary adjustments.

## Materials and Methods

**Fig 1:**
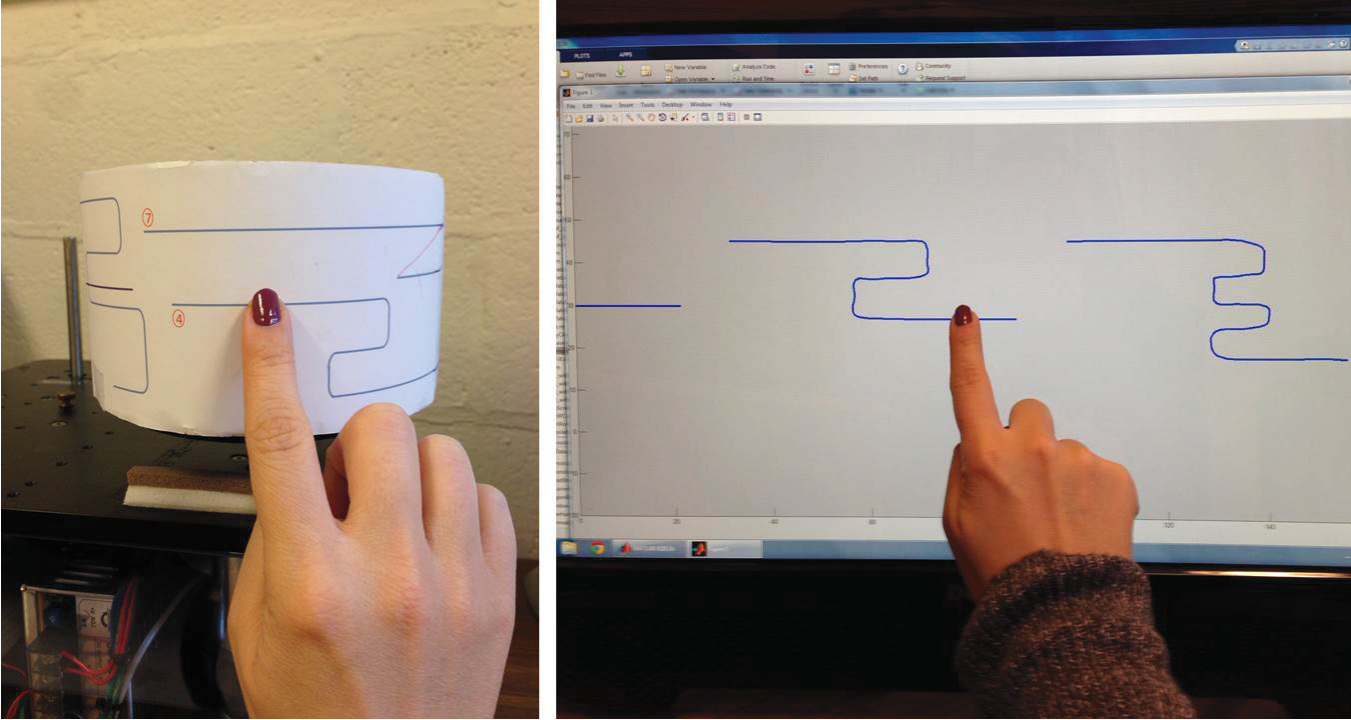
Experimental Setups for ME and TE. Picture depicts the research personnel tracking the curves presented on the surface of the rotating wheel (on the left picture) and on the touchscreen monitor (on the right picture).

We first designed a task in which subjects are asked to track some curves drawn on a paper wrapped around a disc that either rotates at a constant speed or at speeds adaptive to the subject’s horizontal finger motion. This setup (see left picture on Fig 1), which is analogous to rotating wheel Braille display, enables us to evaluate FMAA. On the other hand, in this setup, it is not possible to measure the absolute position of the finger on the curves. Hence, another setup (see right picture on Fig 1) was also designed to study FMAA, this time in digital setting, namely, on a touchscreen monitor where the absolute position of a subject’s finger can be accurately recorded. In all the experiments, subjects are shown some curves that flow from right to left, and are asked to track as many curves as they can while maintaining accuracy by keeping their fingers on the curves.

Since our experiments are inspired by Braille reading, subjects’ performance in these experiments are evaluated based on the metrics used to assess Braille reading speed, which is measured as words per minute with accuracy [39–41]. To accomplish this connection, the following steps were taken in the design of the experiments. First of all, the experiments here are designed for sighted people, hence a curve tracking task is created to encourage subjects to move their fingers from left-to-right and right-to-left directions with the aim to respectively emulate forward reading and reversals observed in Braille reading. Each curve is thought as a Braille sentence, and the performance in the experiments with the disc (see Fig 1) is evaluated by the number of curves that are completely tracked from left to right. Curves are kept in appropriate lengths to be consistent with words per minute measure in Braille reading, so that subjects’ finger motion is continuous on a curve, again similar to what is known in Braille reading that readers do not lose contact with Braille letters even during reversals [22].

Accuracy is another important factor in measuring Braille reading speed [40–42], since it is associated with text comprehension. We also use accuracy as a measure in the experiments with the touchscreen monitor, where we are able to record finger position of the subjects. The accuracy metric here, measured as lateral deviations from horizontal parts of the presented curves, is calculated based on how closely a subject’s finger has completed tracking these curves. This measure helps to penalize a subject’s performance when the subject tries to complete more tasks in a given time at the cost of sacrificing accuracy.

### System

The hardware used in the experiments are an industrial mechatronic drive unit by Quanser and the ST2220T 21.5” multi-touch monitor by DELL. The experiments carried out with the Quanser DC-motor and the touchscreen are referred to as mechanical experiment (ME) and touch screen experiment (TE), respectively, throughout the paper.

#### Mechanical Experiments (ME) with DC-Motor

**Fig 2:**
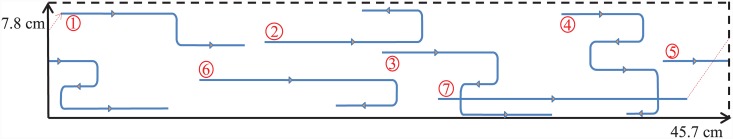
The curve path used in ME. For different experimental conditions the sequence of the curves are changed in order to prevent subjects from memorizing the order. The curve path is printed on a paper and wrapped around the rotating disc. Curves are given in the same aspect ratio as they are seen on the rotating wheel. This figure is 1:2 reduced in dimension to fit the margins. As the wheel rotates clockwise, curves flow from right to left. Each curve is designed such that tracking the curve with the index finger imitates reading of a short Braille sentence. The parts of the curve with arrows pointing left mimic reversals observed during Braille reading. Subjects start tracking with the first curve (marked with red circle), and proceed with the next one. After completing seven distinct curves, they repeat tracking the same path. The red arrow at curve 7 guides the subject to proceed to curve 1, to repeat the path.

The goal in ME is to track with the finger as many curves presented on the rotating wheel as possible within the given time of 45s. Duration of the task is determined such that subjects complete the task without losing interest, or encountering fatigue. The curve path drawn on the wheel is shown in Fig 2 at 1:2 scale with the original aspect ratio. As discussed in the previous section, left-to-right and right-to-left movements of the finger along the designed curves here are inspired from those found in Braille reading, where the parts of the curves folding backwards are considered analogous to reversals. In order to design the length and frequency of these parts, the study in [22] is investigated, where it is reported that reversals are observed often during Braille reading, almost in each sentence. The results of the cited study show that reversals ranged from 0.5cm to 7cm for the sentences used in those experiments. In light of this, in our experiments, we chose the length of the reversal-analogous parts of the curves around the average of these two values (3±2cm), and the frequency approximately as 16 of the total length of the curve path along the horizontal direction. Since during Braille reading activity, words and sentences are read sequentially in a given order, curves in ME are located on the paper such that they can be tracked only sequentially. Subjects are provided help via sequential numbers on each curve indicating the tracking order. Each experimental condition has different sequence of curves to prevent learning.

Subjects take the following two experimental conditions in balanced order, each preceded by a short training session to familiarize the subjects with these conditions:

- **No finger motion adaptive algorithm (NAA)**: The wheel rotates clockwise at a constant speed. A controller is used to keep the speed constant during the experiments. This is especially necessary when subjects touch the disc.
- **Finger motion adaptive algorithm (AA)**: In the absence of finger contact with the wheel surface, the wheel rotates clockwise at the same speed as in the NAA. When the finger touches the wheel, the wheel speed is controlled based on the direction of the finger motion relative to the disc, as applied with normal pressure on the paper. Speed is increased upon the subject’s intention to track upcoming curves faster by moving his/her finger upstream of curve flow, and decreased when the subject moves downstream. The algorithm also lets the subject to reverse the direction of rotation if the curve-path needs to be rewound back.

The speed control of the wheel is implemented based on the disturbance adaptive controller as explained in our previous study in [26] where the controller was tested on a mechanical setup without any human subjects experiments. In the study here, the controller proposed in [26] is used to adapt the wheel speed to the finger motion of human subjects. Specifically, the controllers used here are able to regulate disc speed by sensing how much assistive/resistive torque is being exerted on the disc by the finger. One part of the controller has a predictor to sense this torque and its direction, and the other part regulates the voltage sent to the motor so as to regulate speed as demanded.

Based on pilot studies, a curve flow speed of 6 cm/s, corresponding to 8 RPM of the disc is found to be comfortable enough to accomplish the given curve tracking tasks, and is therefore chosen as the reference speed in the experiments. Higher speeds were avoided as this would cause subjects to track the curves only partially in NAA since subjects would not be able to control the disc speed. Subjects’ performance denoted by *P*_*ME*_ is determined by the number of curves tracked *completely* from the beginning to the end of each curve. Since it is impossible to measure finger position in ME, performance is solely based on this metric.

#### Touchscreen Experiments (TE)

**Fig 3:**
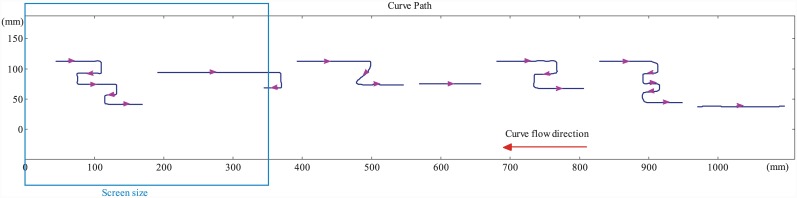
The curve path used in TE. For each condition the sequence of the curves are changed in order to prevent the subject from memorizing the order. The curve path repeats itself with the same sequence until the subject completes tracking within the given time. Curves are given in the same aspect ratio as they are seen on the touch monitor. This figure is 1:4 reduced in dimension to fit the margins. Curves flow from right to left. As in ME, each curve is designed such that tracking the curve with the index finger imitates reading of a short Braille sentence. The parts of the curve that point to left mimic reversals in Braille reading.

In TE, the goal of the subjects is to track as many curves as possible with sufficient accuracy within the given 90s time. Curves are displayed on a touch monitor which enter the screen from right hand side and flow toward left. Fig 3 shows the curve path with a scale of 1:4, keeping the original aspect ratio. The curves are displayed to the subjects within the window shown in the figure, fixed with respect to the screen, and whose size was determined so that the complete body of the curves fit inside it. The curves are not numbered as in ME, since there is no space restriction on the touch screen, and curves flow sequentially. Also, since finger strokes are continuously captured, subjects are allowed to release their fingers from the screen and re-touch/re-track the same curve. This is because, in TE, we have the flexibility to record finger positions in real time. The order of the curves and reversals are designed using the same guidelines as in ME.

Four experimental conditions are tested with the subjects as explained next, with each condition being preceded by a brief training session, and the first two conditions in balanced order:

- **No finger motion adaptive algorithm (NAA)**: Predefined curves flow on the screen of the touch monitor at a constant speed from right to left. FMAA is inactive.
- **Finger motion adaptive algorithm (AA)**: Unless there is contact with the screen, curve flow is at the same speed as in NAA. When the subject traverses the finger on a curve from left to right, then the speed of the curve flow is increased appropriately, enabling the subject to cover the curves faster. As the finger approaches to a corner on the curve, the speed is reduced back so that accurate tracking can be maintained while changing the direction of finger motion. Speed reduction also occurs when the subject wants to move on parts of the curve that point to the left, analogous to reversals.
- **Finger motion adaptive algorithm with increasing speed (AA+)**: Finger strokes of some subjects in AA reached far right of the screen, which caused those subjects to wait for the upcoming curves. In order to provide subjects an opportunity to catch the curves toward the middle of the screen, thereby to be more efficient in performing the tasks, in AA+ experiments, the base speed of the curve flow was made variable within FMAA; it was automatically increased if the finger strokes reached the right half of the screen.
- **No finger motion adaptive algorithm, increased speed (NAA+)**: The average speed of the condition AA+ is calculated for each subject, and then is set as the new constant baseline speed. FMAA is inactive. This condition is identical to NAA except it is performed at a higher baseline speed specifically selected for each subject.

**Fig 4:**
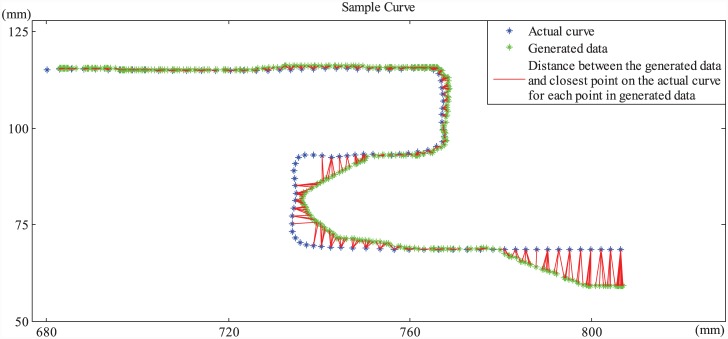
Evaluation of subjects’ performance in TE. Figure represents a sample curve and a trace path generated by the researcher. The actual points of the curve are colored blue whereas the generated trace path/points are seen in green. In order to evaluate subjects’ performance in TE, two metrics are studied: the accuracy and the percentage of the tasks accomplished within given time. Using a nearest point search algorithm, the closest points on the actual curve for each point in the subject’s trace path are found. The sample in the figure shows the distances between those closest points and the tracked path colored in red. A good performance is represented in the first upper half of the curve where the distance between the closest points and user points are relatively shorter indicating that the user was able to track the path as close to the actual path as possible.

FMAA used in TE consists of two parts: a position controller and a real-time nearest point search algorithm available in MATLAB (The MathWorks), as explained in the Appendix. The performance of a subject in TE is measured using two metrics *P*_*TE*_1__ and *P*_*TE*_2__, determined respectively based on what percentage of the curves the subject is able to cover within the given time and how accurately the curves are tracked. In order to calculate the coverage percentage, nearest point search algorithm is used to find the points on the actual curve that are the closest to the path tracked by the finger (see Fig 4). The closest points on the actual curve provide a measure of how closely the subject is able to track the actual curve. *P*_*TE*_1__ is calculated by the ratio of the number of unique closest points to the total number of points on the actual curve path. In order to calculate *P*_*TE*_2__, which is the normalized error, the total distance between the closest points and the tracked path is divided by the number of closest points.

### Ethics Statement and Participants

The study was approved by the Institutional Review Board at Northeastern University (IRB Protocol Number: 13-08-32). Experiments are announced through flyers that were hung around Northeastern University campus. The experiments were open to volunteering sighted and healthy subjects who were at least 18 years old with sufficient literacy level in English. No compensation is paid to subjects for their participation in the experiments. Prior to the experiments, written consent was obtained from each participant. The experiments are carried out with thirty sighted subjects among whom there were 12 females and 18 males with ages ranging from 23 to 33 years.

### Protocol

After obtaining written consent, participants are asked to sit comfortably in front of the rotating wheel and touch monitor in ME and TE, respectively. They are instructed to use their index finger of their dominant hand to follow the curves, and rest their elbows on the table in case their arm gets tired. For ME, they are asked to move their fingers only in their field of view without leaning over to forecast the upcoming curves. The goal in the experiments is explained to the subject. A training session is completed by the subject prior to each experimental condition. The curve paths given in the training sessions are different than the curve paths used in the actual experiments. In both ME and TE, in order to study the effect of order of different experimental conditions, half of the subjects are given the NAA first whereas the other half starts with condition AA. Conditions AA+ and NAA+, when applicable, are taken subsequently (details below). Moreover, the sequence of the curves is different in each condition to prevent learning. Each subject is given a NASA TLX survey after completing each condition, where subjects evaluated mental, physical, temporal demands, performance, effort and frustration by a score from 0 to 100. A questionnaire is completed by each subject after finishing the whole experiment. The questionnaire is aimed to determine if the subject had any issues adapting the AA condition and if s(he) had any recommendations on FMAA. For TE, subjects are also asked to fill out a motion sickness questionnaire before and after the experiments. In ME, subjects’ hand movements are recorded with a camera upon their consent. These recordings/videos are used for the evaluation of subjects’ performance as a group.

### Statistical Analysis

Statistical analysis is performed to study any significant differences in performance metrics between experimental conditions in ME and TE. The analysis is carried out using SPSS.

In ME, the difference scores *P*_*ME*_ were normally distributed, as assessed by Shapiro-Wilk’s test (*p* = 0.396). Two-tailed paired-samples t-test is used to analyze statistical significance between conditions AA and NAA.

In TE, curve coverage performance scores *P*_*TE*_1__ for conditions NAA, AA, AA+ and NAA+ were not normally distributed as evaluated by Shapiro-Wilk’s test (*p* < 0.05, for score samples in all conditions). Since data transformation did not succeed in normally distributed samples, a non-parametric method alternative to the repeated measures ANOVA test, namely the Friedman test, is used for statistical analysis. Post-hoc comparison tests are performed with a Bonferroni correction for multiple comparisons. Error samples, *P*_*TE*_2__ are also found to have non-normal distributions for all conditions (*p* < 0.05, Shapiro’s Wilk). One-way repeated measures ANOVA test is used to determine any statistical significance in *P*_*TE*_2__ between all conditions. *P*_*TE*_2__ samples are log transformed to meet the normality and sphericity assumptions of the repeated measures ANOVA test. Normality and sphericity has been validated by Shapiro-Wilk’s and Mauchly’s tests, respectively. Post-hoc comparison tests with Bonferroni adjustment are performed to reveal any statistical significance between pairwise combinations of all conditions. As analyses were performed in logarithmic scale, results are reported as back-transformed means (geometric mean) with 95% confidence intervals.

In order to reveal any statistical significance in NASA TLX scores between different conditions, a two-tailed paired-samples t-test is performed in ME, and one-way repeated measures ANOVA tests are used in TE, where pairwise comparisons are evaluated by post-hoc tests with a Bonferroni correction.

The significance level for all statistical tests is set to *p* < 0.05. Values reported in the next section with ± represent mean ± standard error. All raw data including video recordings can be accessed at [43].

## Results

### FMAA helps to accomplish more tasks per time in ME

Subjects’ scores in ME are presented on Table 1. The values presented under *attempt* and *success* for conditions NAA and AA indicate how many curves subjects started tracking, and how many of these they completed successfully. Both conditions correspond to a success rate around 95%, where 5% failure rate is due to the fact that, within the given time, the last attempted curve was not completed.

**Table 1:** Performance in ME and Reversal Completion Rate in TE.

	ME								TE	
	Curve Coverage								Reversal Completion Rate	
	NAA			AA			*P*_*ME*_			
	# of curves		%	# of curves		%				
Subject #	Attempt	Success		Attempt	Success		NAA	AA	AA	AA+
**S**_**1**_	8	8	100	13	12	92	47.1	70.6	94	91
**S**_**2**_	8	8	100	13	12	92	47.1	70.6	92	91
**S**_**3**_	8	8	100	12	11	92	47.1	64.7	96	97
**S**_**4**_	8	7	88	10	9	90	41.2	52.9	97	94
**S**_**5**_	8	8	100	15	15	100	47.1	88.2	98	85
**S**_**6**_	8	7	88	12	12	100	41.2	70.6	97	94
**S**_**7**_	8	8	100	13	13	100	47.1	76.5	98	90
**S**_**8**_	8	8	100	11	10	91	47.1	58.8	95	93
**S**_**9**_	8	8	100	14	13	93	47.1	76.5	96	99
**S**_**10**_	8	7	88	14	14	100	41.2	82.4	97	93
**S**_**11**_	8	7	88	14	13	93	41.2	76.5	86	83
**S**_**12**_	8	8	100	11	11	100	47.1	64.7	86	78
**S**_**13**_	9	8	89	18	17	94	47.1	100	87	85
**S**_**14**_	8	8	100	15	15	100	47.1	88.2	81	80
**S**_**15**_	8	8	100	16	16	100	47.1	94.1	96	86
**S**_**16**_	9	8	89	14	13	93	47.1	76.5	98	90
**S**_**17**_	9	8	89	13	11	85	47.1	64.7	98	94
**S**_**18**_	9	8	89	13	12	92	47.1	70.6	90	83
**S**_**19**_	8	8	100	12	11	92	47.1	64.7	95	89
**S**_**20**_	8	8	100	10	10	100	47.1	58.8	87	83
**S**_**21**_	9	8	89	15	14	93	47.1	82.4	94	86
**S**_**22**_	9	8	89	13	12	92	47.1	70.6	82	88
**S**_**23**_	8	8	100	10	10	100	47.1	58.8	90	85
**S**_**24**_	9	8	89	9	8	89	47.1	47.1	86	86
**S**_**25**_	9	8	89	14	14	100	47.1	82.4	95	90
**S**_**26**_	9	8	89	10	10	100	47.1	58.8	92	87
**S**_**27**_	9	8	89	12	11	92	47.1	64.7	76	71
**S**_**28**_	9	8	89	15	14	93	47.1	82.4	84	85
**S**_**29**_	9	8	89	14	13	93	47.1	76.5	90	89
**S**_**30**_	9	8	89	18	17	94	47.1	100	89	88
Mean	8.4	7.9	93.5	13.1	12.4	94.9	46.3	73.1	92	88
Std. error	0.1	0.1	1.1	0.4	0.4	0.8	0.4	2.4	1.1	1
t-test	p<0.0005								

The performance score *P*_*ME*_ indicating the task completion percentage is calculated by normalizing the number of successfully tracked curves where the subject among the 30 that achieved tracking of the maximum number of curves is given the maximum score of 100 points. The comparison of *P*_*ME*_ between NAA and AA shows that subjects completed 1.6 times more tasks in AA when FMAA was active. The two-tailed paired-samples t-test shows that task completion percentage *P*_*ME*_ in AA with the mean of normalized score 73.1±2.4% is significantly higher than in NAA with the mean of normalized score 46.3±0.4%, with an increase of 26.83%(*t*(29) = 11.15, *p* < 0.0005, *d* = 2.04).

**Fig 5:**
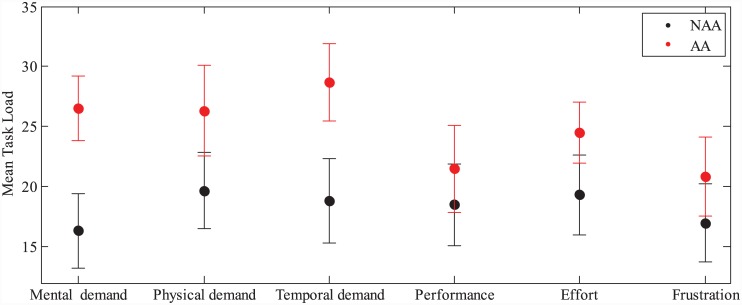
Mean workload metrics for different experimental conditions performed in ME. Results are based on NASA–TLX. Error bars represent standard error. Mental demand scores in NAA and AA are found to be statistically significant at the 0.05 level while no statistical significance is observed for the remaining workloads.

Fig 5 shows the NASA TLX results for conditions NAA and AA where the mean load for each task is higher in AA. While the mental demand is significantly higher in AA by a score of 10.2±3.6 when compared to NAA (two-tailed paired-samples t-test, *t*(29) = 2.8, *p* = 0.008), variations in other task load metrics do not permit to draw any statistical significance.

### FMAA is used effectively for increased curve coverage in TE

**Fig 6:**
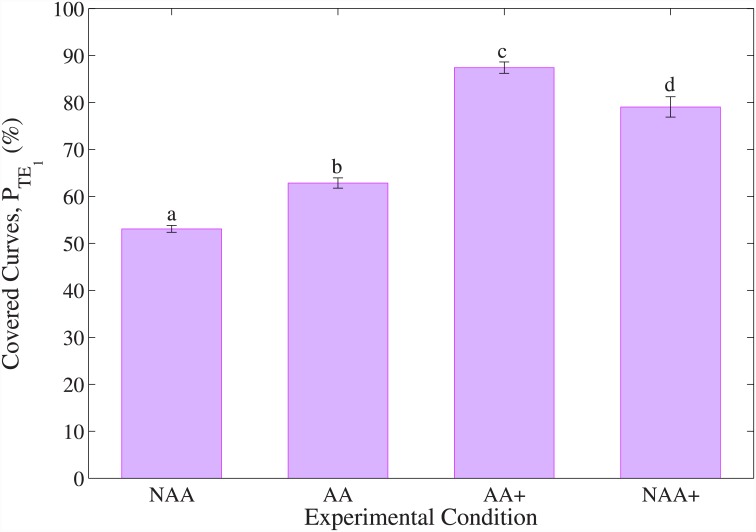
Median percentage of the curve path covered by the subjects for each experimental condition in TE. Error bars represent the standard error. Different letters on error bars indicate statistical significance in pairwise comparisons performed with Bonferroni post-hoc tests.

**Fig 7:**
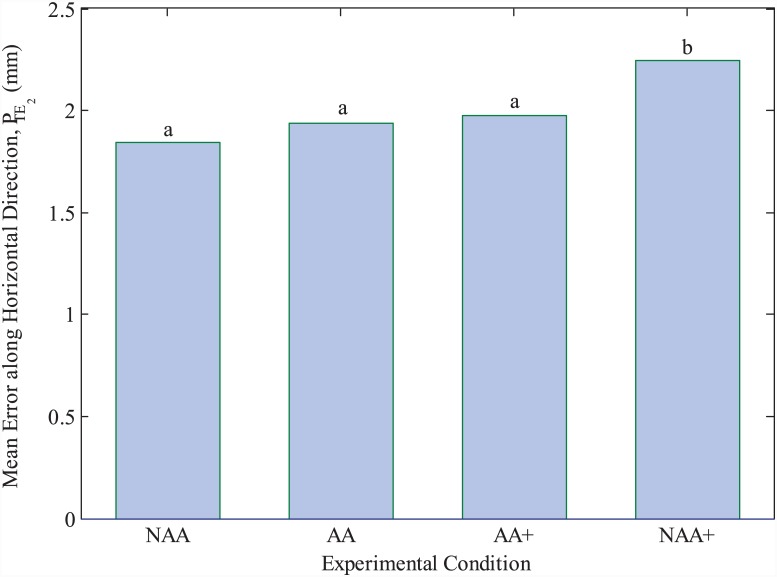
Mean lateral deviations from the horizontal parts of the curve paths in TE. Mean values are geometric means calculated by antilog of transformed data. Different letters on bars indicate statistical significance in pairwise comparisons performed with Bonferroni post-hoc tests. See ‘Results’ section for confidence intervals.

Results from TE are presented in Figs 6 and 7. Fig 6 shows the mean curve-coverage-percentage *P*_*TE*_1__ for different experimental conditions. In order to calculate *P*_*TE*_1__, the highest achieved curve-coverage-percentage is found among the four conditions (NAA, AA, AA+, NAA+), and is taken as 100 points, and all the remaining scores are normalized based on this value. Statistical analysis is performed using the non-parametric related-samples Friedman’s test due to the non-normal distribution of *P*_*TE*_1__ scores, as assessed by Shapiro Wilk’s test (*p* < 0.05 for all conditions), and failed data transformation. Curve-coverage-percentage was statistically significantly different between different conditions (Friedman’s test, *X*^2^(3) = 79.56, *p* < 0.0005). Post-hoc analysis is performed with two-tailed Bonferroni correction for multiple comparisons, and revealed statistically significant differences in *P*_*TE*_1__ scores from NAA (*Mdn*±*SE* = 54.6±0.7%) to AA (*Mdn*±*SE* = 63±1.1%) (*p* = 0.016), as well as from NAA+ (*Mdn*±*SE* = 79.6±2.2%) to AA+ (*Mdn*±*SE* = 88.8±1.2%) (*p* = 0.031). When FMAA is used with the increased base speed setting (AA+), subjects performed the highest scores, with significant differences in comparison to other experimental conditions (two-tailed Bonferroni correction for multiple comparisons, between NAA and AA+ (*p* < 0.0005), and between NAA+ and AA+ (*p* = 0.031)).

### FMAA is used effectively without losing accuracy in TE

A one-way repeated measures ANOVA was conducted to determine whether there were statistically significant differences in *P*_*TE*_2__ metric, between experimental conditions NAA, AA, AA+ and NAA+. *P*_*TE*_2__ values were not normally distributed (Shapiro Wilk’s test, *p* < 0.05 for all conditions). Hence, normality was achieved by log-transformation of the data. Sphericity assumption was met as assessed by Mauchly’s test, *X*^2^(5) = 8.62, *p* = 0.125. One-way repeated measures ANOVA test is performed on the log-transformed data and showed statistically significant changes in *P*_*TE*_2__ between conditions NAA, AA, AA+ and NAA+, *F*(3,87) = 11.46, *p* < 0.0005, partial *w*^2^ = 0.35. Fig 7 presents the mean lateral deviations of subjects’ fingers from the horizontal parts of the actual curve path, which are calculated as 1.84mm (95% CI, 1.65 to 2.05mm), 1.94mm (95% CI, 1.75 to 2.15mm), 1.97mm (95% CI, 1.8 to 2.16mm) and 2.24mm (95% CI, 2.05 to 2.5mm) for NAA, AA, AA+ and NAA+ conditions, respectively. Notice that the means presented in Fig 7 are calculated by the anti-log of the means of the log-transformed data, therefore representing the geometric means of the original data. Pairwise comparisons between conditions are presented next, which are performed using post-hoc tests with a Bonferroni adjustment. The increase of 0.1mm in *P*_*TE*_2__ from NAA to AA is found to be not statistically significant (*p* = 0.446), as well as the increase of 0.13mm from NAA to AA+ (*p* = 0.242). In other words, such deviations from the horizontal segments of the curves were not significantly different with increased average curve flow speeds in AA+ when compared to lower speeds in NAA and AA. However, accuracy dropped significantly as the curve flow speed is increased in the absence of FMAA in condition NAA+, where the mean of *P*_*TE*_2__ increased significantly by 0.27, 0.3 and 0.4mm when compared to NAA (*p* < 0.0005), to AA (*p* = 0.012), and to AA+ (*p* = 0.01). Since the vertical parts of the curves are used only to connect the horizontal segments, performance along vertical segments of the curves was not of interest, and was found to not show any statistical significance.

### Subjects perceived increased mental demand with FMAA

**Fig 8:**
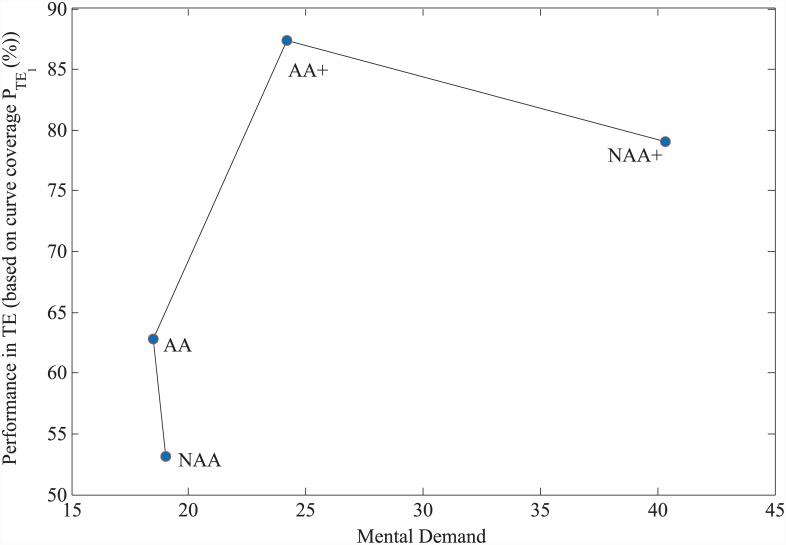
Average Mental Demand vs. Performance graph for TE based on NASA–TLX. Performance of subjects in TE experiments based on curve coverage versus mental demand as measured by NASA–TLX, where error bars represent the standard error. See Fig 6 for significance of this performance metric.

**Fig 9:**
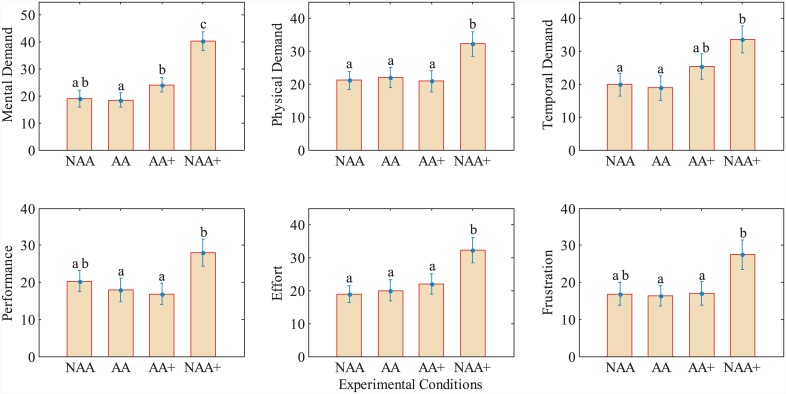
Mean workload metrics for different experimental conditions performed in TE. Results are based on NASA–TLX. Error bars represent standard error. Different letters on bars indicate statistical significance in pairwise comparisons performed with Bonferroni post-hoc tests. Letters shared in common between experimental conditions indicate no significant difference.

For each task, subjects evaluated mental, physical, temporal demands, performance, effort and frustration via NASA TLX, where a workload score is ranged from 0 to 100. As seen in Fig 8, mental demand was lower in experimental conditions AA (18.5±2.6) and NAA (19±3.1) than in conditions AA+ (24.2±2.6) and NAA+ (40.3±3.4). With increased mental demand in AA+, the performance reaches higher value but noticeably decreases under the condition NAA+ in which the mental demand is the highest and the performance is the poorest based on *P*_*TE*_1__ and *P*_*TE*_2__. A one-way repeated measures ANOVA test revealed statistical significance in mental demand between conditions NAA, AA, AA+ and NAA+ (*F*(3,87) = 25.5, *p* < 0.0005). Pairwise comparisons with a Bonferroni correction showed that the increase in mental demand from NAA, AA and AA+ to NAA+ is statistically significant (for all comparisons *p* < 0.0005), as well as from AA to AA+ (*p* = 0.045). Physical demand (PD), temporal demand (TD), performance (PF), effort (EF) and frustration (FR) are also found to be statistically significant between different conditions by one-way repeated measures ANOVA tests (PD: *F*(3,87) = 6.4, *p* = 0.001, TD: *F*(3,87) = 6.14, *p* = 0.001, PF: *F*(3,87) = 6.54, *p* = 0.0005, EF: *F*(3,87) = 8.23, *p* = 0.001, FR: *F*(3,87) = 5.97, *p* = 0.001). Post-hoc tests with a Bonferroni correction for pairwise comparisons revealed that mental demand, physical demand and effort are significantly higher in NAA+ when compared to NAA, AA and AA+ (for all *p* < 0.01). The remaining three workloads, namely, temporal demand, performance and frustration showed statistical significance in some pairwise comparisons as reported in Fig 9.

## Preliminary Results on the Evaluation of FMAA by Blind Participants

Upon validating successful operation of FMAA with sighted people, we next carried out preliminary experiments with six blind subjects under approved IRB. Below we present our early findings.

### Materials and Methods

**Fig 10:**
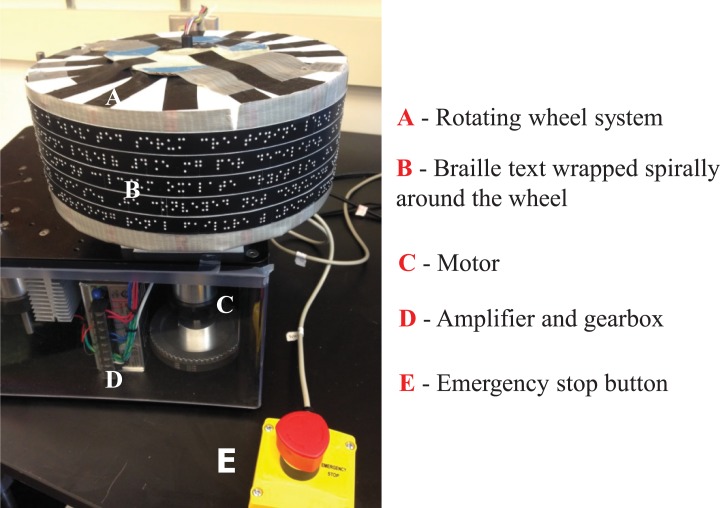
Experimental setup used in the human subject testing with blind individuals. Setup components marked on the picture are explained on the right hand side with matching letters.

In the experiments, the task presented to subjects is reading passages that are embossed in Braille using a continuous labeler tape and wrapped spirally around a rotating wheel (see Fig 10), which either rotates based on FMAA, or at a constant speed. This constant speed corresponds to each subject’s comfortable Braille reading speed. Braille texts are embossed using a Braille labeler. On the labeler, contracted forms of commonly used words are readily available. Among those we have used *for, the, and, of, to* and *with* in the text while the remaining words were embossed in uncontracted form, which permitted a more transparent analysis of reversals from prerecorded videos.

With the experiments, we aim to explore whether or not the rotating wheel system would properly respond to subject’s finger motions in the presence of FMAA to accommodate subject’s forward and reversal finger motions, and how these results compare with Braille reading without the FMAA. In order to investigate this, in the experiments we used two different texts T_1_ and T_2_, which are borrowed from a medical journal [44]. Some of the words in the texts are replaced with low-frequency words to encourage reversals, see [43]. The degree of complexity of the phrases T_1_ and T_2_ are made comparable by balancing the percentage of low-frequency words in both texts. The frequency data is checked based on [45] using The Corpus of Contemporary American English, which indicated both texts to have 41% low frequency words. In all the experiments, subjects are asked to read the presented text out loud with accuracy, without having to focus on the reading speed. A subject’s performance in the experiments is hence evaluated based on how accurately the subject completes reading the given text.

### System

The setup used in the experiments with sighted people is used with modifications on wheel size for larger reading space, and information displayed on the wheel surface is now Braille texts, T_1_ or T_2_ (see Fig 10). Also some technical modifications on FMAA and tuning of the controller parameters were required in order to optimize the algorithm for the use by the blind people, see the technical details and initial piloting studies in [46].

### Participants

In order to recruit blind subjects, we have collaborated with the National Braille Press (NBP). Subjects that participated in the experiments are proofreaders and can be considered as advanced Braille readers as they have at least 20 years of Braille reading experience. Experiments are performed with 4 female and 2 male subjects whose ages ranged from 24 to 65. No compensation is paid to the subjects for their participation in the experiments.

### Protocol

After obtaining subjects’ consent, experimental protocol and goals are explained to each subject by the research personnel. While reading Braille on the rotating wheel system, subjects interacted with the Braille text presented on the wheel surface by holding their reading hand about 45° above their viewplane. Subjects expressed comfort in using a single finger for Braille reading, and in the experiments, they used a single finger in their dominant hand to read Braille. Before starting reading, subject’s finger is positioned in the beginning of the text. Training sessions are completed by the subject prior to experiments. Subjects were encouraged to take as many training sessions as they needed until they felt comfortable with reading Braille on the rotating wheel. However, research personnel paid attention not to exceed the time devoted for the experiments as reported in the consent form. On the average training sessions took about 13 min. After completing the training session, subjects respectively took the experiments with FMAA (with text T_1_), and without FMAA (with text T_2_). Experiments ended when subjects completed reading the given text. Their hand motions are recorded during experiments by the research personnel without revealing their identity. Before and after the experiments, subjects are asked some survey questions to evaluate their general comfort and subjective assessment on the efficacy of FMAA.

### Training and Experiments

Prior to experiments, each subject’s average Braille reading speed *v*_*avg*_ is measured in cm/s. For this, the subject reads the first two paragraphs of the New York Times article in [47], which consists of 91 words corresponding to 360cm length, and embossed in Braille on thick Braille paper. This speed *v*_*avg*_ is then used as the baseline speed in the following experiments with texts T_1_ and T_2_.

Before reading the texts T_1_ and T_2_, each subject takes training sessions to practice reading Braille on the rotating wheel with and without FMAA, as described in Protocol. The text given to subjects in the training session consists of mostly simple sentences and also some numbers, pseudowords and low-frequency words. In the start of training session, the wheel speed is set to the angular speed rendering a linear speed of *v*_*avg*_/2. It is then gradually increased while communicating with the subject about his/her comfort in reading. The final speed adjusted based on the needs of the subject is used in the subsequent experiments as the baseline speed *w*_*bl*_.

Following the training session, similar to ME experiments performed with sighted people, subjects took two conditions sequentially where in both conditions wheel speed was set to the baseline speed *w*_*bl*_ determined as described above. For the first experimental condition, each subject is given the text T_1_ and FMAA is active, allowing variable reading speed based on finger motions of the subject. In the second condition the wheel speed is kept constant without FMAA and subjects are presented the text T_2_. There is about five minutes setup time when replacing T_1_ with T_2_. During experiments, subjects’ finger motions are captured with a camera upon their consent. Subjects’ performances are evaluated based on what percentage of the text they have read accurately out loud, which is calculated by dividing the number of accurately read words by the total number of words displayed in the text. This post experimental analysis is carried out with careful study of the recorded videos, which are available upon request to the corresponding author.

### Results

**Fig 11:**
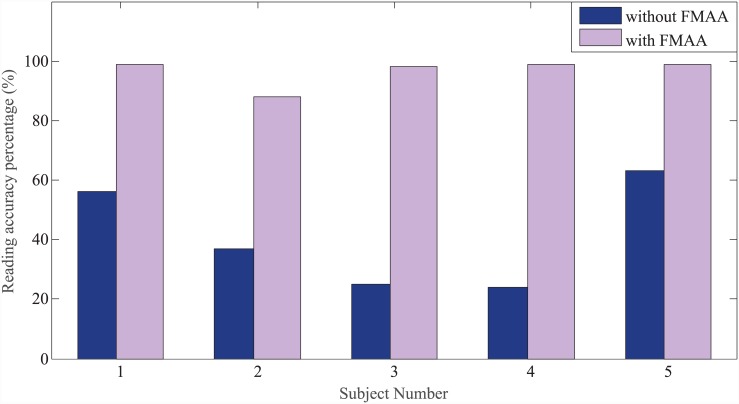
Braille reading accuracy in different experimental conditions. Percentages shown in the figure are calculated by dividing the number of accurately read words by the total number of words in each experimental condition. Accurate reading is assessed based on subject’s reading out loud.

Preliminary experimental results are presented in Fig 11, which illustrate subjects’ reading accuracy performances for different experimental conditions. Results reflect performances of five subjects, as one of the subjects chose not to proceed to the experimental phase. While FMAA was active, subjects on average read 96.6% of the given text accurately, whereas without FMAA their reading accuracy on average was 41%. Based on these results, when we compare subjects’ performances between experimental conditions with and without FMAA, where each given text is introduced to the subjects for the first time, we observe that with FMAA subjects accomplished the Braille reading task with much higher performance based on the predefined goal. The efficacy of FMAA is evaluated next by the subjects with an average score of 7.6/10, with 10 representing the most effective.

Subjects’ feedback on the rotating wheel system and the efficacy of FMAA in Braille reading was as follows. Some subjects suggested to also test FMAA with contracted Braille, which can further improve subjects’ reading ability. Further, subjects mentioned that they found backward rewinding of the text adaptive to their finger motions very helpful in terms of being able to rescan any word/part of the text when they needed to reread it. Subjects reported slight arm pain after the experiments due to holding up their hand during Braille reading. They recommended positioning the rotating wheel horizontally on a flat surface such that they can locate and use their hands during Braille reading as they do with conventional Braille displays or paper.

## Discussion

In this study, a finger motion adaptive algorithm (FMAA) is proposed, tested, and evaluated with sighted and blind subjects. Based on the experimental results and statistical analysis with sighted subjects, we have the following observations and discussions:

ME results show that when curve flow speed related to wheel rotating speed varied adaptive to subject’s finger motion, rather than being at a constant value, subjects were able to track more curves within the given time. Since in AA it is possible to reduce the curve flow speed on reversals, and even reverse the curve flow direction if needed, it was unknown to us whether or not subjects would be able to complete as many tasks as they could in NAA. However, our findings show that, with little training, subjects used FMAA effectively in AA in a way to compensate for the time they lost at lower speeds, by accelerating the curve flow whenever they were comfortable.

One may argue that the reason subjects performed poorer in NAA in ME is that the pre-set constant curve flow speed for NAA is too high which may have caused subjects to fail tracking some of the curves. This is however not the case as seen from the success/attempt rates on Table 1 for both AA and NAA conditions, where subjects were able to successfully complete approximately 95% of all the attempted tasks in both conditions. This indicates that the constant speed in NAA as well as the base speed in AA were convenient for the subjects to complete their tasks.

When we inspect the performance levels *P*_*ME*_ and the subjective workload assessment presented respectively on Table 1 and in Fig 5, we observe that the performance and the mean mental workload are both increased in the presence of FMAA. An interpretation of this increase can be that subjects were more engaged and alert with the tasks when FMAA was active, causing increased attention and thus relatively higher mental workload which in turn helped subjects concentrate and perform better in AA. In other words, mental workload increase was sufficient enough to guide the users to increased performance, without overwhelming them in their tasks.

Although ME results indicate that subjects accomplished more tasks using the finger motion adaptive algorithm, we wanted to further investigate whether the accuracy along the tracked path has dropped significantly in the presence of FMAA due to temporally increasing speeds. For this, we designed similar tasks but to be performed on a touchscreen monitor so that we could record finger positions of the subjects. The results obtained from TE were consistent with ME results, showing that subjects covered a higher percentage of curves when FMAA was active. The accuracy in the presence (AA) and absence (NAA) of FMAA did not differ significantly although AA on average had a higher curve flow speed than NAA.

While analyzing the results for NAA and AA, we noticed that the recorded finger positions of some subjects concentrated on the far right of the screen. This indicated that at some time instants, subjects waited for the upcoming curves to enter the screen, which indicated inefficiency, and raised the question of whether or not subjects would be able to track the curves at a speed higher than the given base speed. In order for the subjects to catch the curves further to the middle of the screen, we designed another experimental condition AA+, a modified version of AA, where the initial curve flow speed is the same as the base speed in AA, but is increased if the finger position of the subject starts to settle toward the far right of the screen. In AA+, we tested whether the subjects would be able to take advantage of FMAA and cover a higher percentage of curves at a higher base speed than in AA and NAA. We also inspected whether there would be significant loss in accuracy along the horizontal direction due to higher base speed. After analyzing the results of AA+, we found that subjects performed significantly better in AA+ than they did in NAA and AA. Accuracy in the horizontal segments of the curves in AA+ did not differ significantly from NAA and AA either. Contrary to our expectation, accuracy did not drop with the increased speed in AA+, but almost remained the same when compared to AA (0.05% difference).

FMAA allows subjects to track curves faster on horizontal lines that are in left-to-right direction, while it automatically reduces the base speed as a reversal is approached so that finger tracking is accommodated. Although subjects are asked to complete tracking of each curve from start to end, and research personnel observed fair play, one concern here might be that subjects may still achieve high scores by tracking the curves only in the left-to-right directions, and skipping the reversals. This concern may exist in TE, since in ME, the performance *P*_*ME*_ is evaluated based on full completion of the curves, without partial credits. To address this in TE, we investigated completion rates on reversals, Table 1. For this, each subject’s ability to touch as many points as possible on reversals are calculated as a ratio to all such points on reversals, up to the particular termination point in the experiments. As seen on Table 1, subjects were able to touch a large portion of the reversals presented to them (92% and 88% of all possible reversal points in AA and AA+, respectively), and hence subjects reflected this in their performance metrics.

After completion of experiments with conditions NAA, AA, and AA+ in a single session, during analysis of the collected data, the need has arisen to investigate whether or not subjects would be able to perform better than in AA+ if they were given the condition NAA but at a higher constant speed. For this, we designed the condition NAA+, and contacted the subjects to request for participation in an additional experiment with the condition NAA+, first preceded by a training session. All the same subjects agreed to participate. In NAA+, the finger motion adaptive algorithm was not active, and the average curve flow speed in AA+ for each subject was calculated separately, and set as the constant curve flow speed in NAA+. The results of NAA+ showed that the performance was significantly lower when compared to AA+, and the accuracy also dropped significantly with respect to the other conditions. When we compare the results of NAA and NAA+, we observe that there is a trade-off between the task completion rate and the accuracy. The increased speed in NAA+ helps subjects complete more tasks when compared to NAA, however, it also causes considerably increased lateral deviations in the horizontal segments of the curves, sacrificing accuracy.

Overall, when we investigate the experimental results, we observe that subjects tend to benefit from FMAA to complete more tasks while maintaining accuracy. This is the case in both ME and TE. With regard to ME, subjects completed 1.6 times more tasks in the presence of FMAA. In TE, when FMAA is used with increased speed setting (AA+), we reached the same performance factor of 1.6 when compared to NAA. This indicates that although the initial base speeds were the same for conditions NAA and AA+, in AA+ subjects took the opportunity to execute the tasks at higher speeds, without sacrificing accuracy in any noticeable way.

Some of the above findings can also be related to well known interplay between performance versus mental workload [25]. In TE, the performance increases with mental demand up to a maximum, and starts to decrease after that point in the presence of much higher mental demand. In Fig 8 mental demand from NASA-TLX surveys is plotted with respect to performance (see also Fig 9), which is also consistent with [25]. In the figure, increased performance and mental demand in AA+ indicates that subjects are more engaged with the tasks in AA+ with positive outcomes, however, they are overwhelmed with too much mental workload in NAA+ which causes them to perform worse in the given tasks. Statistical results clearly support this discussion. Pairwise comparisons with a Bonferroni correction are performed between different experimental conditions for workloads, and, mental, physical demands and effort are found to be significantly higher in NAA+ when compared to NAA, AA, and AA+. These metrics between experimental conditions NAA vs. AA, and NAA vs. AA+ however did not show any statistical significance, while subjects’ performance were significantly higher in AA and AA+, when compared to NAA, as shown in Fig 8.

Based on preliminary experiments with blind subjects, we have the following observations: Although the subjects were not accustomed to reading Braille on a rotating wheel type display, with little training they were able to read the Braille on the wheel. Finger-motion adaptive speeds presented with FMAA helped subjects re-read any words they needed to re-scan for complete decoding of the word. When subjects attempted to move their fingers from right to left to position their finger in the start of the word/words they wanted to re-read, FMAA let the wheel slow down or rewind (rotate CCW) allowing subjects to locate their finger at the desired position easier and faster. On the contrary, in the experiments without FMAA, when subjects needed to re-read a word, they usually either skipped the word without out loud reading or read it partially and inaccurately. The reason that subjects mostly avoided reversals could be that they did not want to lose time on reversals, since this would cause them to miss the subsequent words as they did not have the option to slow down the wheel speed. In summary, subjects achieved higher reading accuracy rates with FMAA, although both experiments with and without FMAA had the same baseline speed determined based on each subject’s comfortable average Braille reading speed.

Future work includes improvements of the rotating wheel and FMAA based on subjects’ feedback, testing with a large number of subjects to show statistical significance of the results, and comparing results with uncontracted and contracted Braille. Preliminary experimental results obtained with blind subjects are very encouraging motivating us to further improve the rotating wheel system.

## Conclusion

This study is inspired from intricate reversal movements of fingers as performed by visually impaired people while reading Braille. Here, feasibility of a finger motion adaptive algorithm (FMAA) is assessed by sighted human subjects in experiments with finger-tracking tasks in a machine/device, analogous to Braille reading and “reversals”. Based on the experimental results and statistical analysis of performance metrics pertaining to task accuracy and task completion, it is found that FMAA has been effectively utilized by the subjects, with little training, to improve these metrics. Furthermore, preliminary experiments are carried out with blind subjects, analysis of which show that FMAA was quite useful in improving Braille reading accuracy. All these results show potential for creating a refreshable and affordable version of the rotating wheel Braille display, in which FMAA can provide users ergonomic and adaptive Braille reading experience.

## Supporting Information

S1 Appendix (PDF)

S1 Table Notations. (EPS)

S1 Fig Geometry of curve path elements. (EPS)
