# Supplementary material for: Experimental Evaluation of a Braille-Reading-Inspired Finger Motion Adaptive Algorithm
Source: PLoS One. 2016 Feb 5;11(2):e0148356. doi: 10.1371/journal.pone.0148356 (PMC4743913; doi:10.1371/journal.pone.0148356)
Supplement: S1 Appendix — (PDF) [file pone.0148356.s001.pdf]

S1 Appendix

Position controller in TE works as follows: When a subject's finger interacts with a curve at a point p_t, two independent position controllers, one in x direction and the other in y direction, are used, with proportional gains denoted by k_{px} and k_{py}. The controller first calculates the traveled distance along x- and y-coordinates relative to the first interaction point p_t, and these distances are introduced to the controller as errors denoted by δx and δy. Then, the horizontal position of the curve path is regulated based on the error δx penalized by the controller gain k_{px} whereas the regulation along vertical direction is performed only on the particular curve that the subject is interacting, and is calculated based on the error δy penalized by the controller gain k_{py}. When the interaction with the touch screen is removed at any instance, then the position controllers are reset to zero, and curve flow speed resumes its baseline value.

The nearest point algorithm is employed to enhance the tracking speed of the subject, see S1 Table for definitions:The curve path is divided into regions R_i via vertical boundaries b_{R_i} not seen by the subject (dashed in S1 Fig.). Each region R_i contains a curve C_i which was created in Matlab as an interpolating cubic spline that consists of points $p_k, k = 1..101$. Each curve C_i is offset by a horizontal distance m. The slope s_{p_k} at each point p_k of the curves is calculated using p_{k+1} in first order Taylor expansion. According to s_{p_k} value, p_k is determined to be either on a vertical segment (if $|s_{p_k}| > 0.34$), or on a horizontal segment of the curve. This range for slopes was determined in pilot studies based on what the subjects in general perceived to be horizontal or vertical finger motions.

The start and end point of each vertical part is considered as a corner c of the curve. Region boundaries $b_{R_{i_l}}$ and $b_{R_{i_r}}$ for each curve C_i are determined such that they are located on the left and right of the curve by an offset of $^m/_2$. For each touched point p_t by the subject, the curve region R_i containing p_t is found in order to determine the active curve C_i that is the closest to the touched point p_t. Then, using the nearest point search algorithm in Matlab, the closest point p_n to the touched point p_t is calculated. This way the error in position tracking can be calculated.

Following action is taken if p_n is on a horizontal part of the curve: The gain k_{px} of the position controller is increased such that the curve flows faster underneath subject's finger. As p_n approaches the corner c of the curve, k_{px} is reduced in real-time to its previous value in order to prevent subject's finger to overshoot the corner of the curve and undesirably deviate from the curve. When p_n is on the vertical part of the curve, the position controller gain k_{py} is increased at each time step up to a predetermined value and kept at that value until the finger reaches a corner on the active curve.
